# Development and analysis of thick GaN drift layers on 200 mm CTE-matched substrate for vertical device processing

**DOI:** 10.1038/s41598-023-42747-1

**Published:** 2023-09-23

**Authors:** Walter Gonçalez Filho, Matteo Borga, Karen Geens, Deepthi Cingu, Urmimala Chatterjee, Sourish Banerjee, Anurag Vohra, Han Han, Albert Minj, Herwig Hahn, Matthias Marx, Dirk Fahle, Benoit Bakeroot, Stefaan Decoutere

**Affiliations:** 1 IMEC-Interuniversity Microelectronics Center, Kapeldreef 75, 3001 Leuven, Belgium; 2 CMST-IMEC, Ghent University, Technologiepark 126, 9052 Ghent, Belgium; 3 Aixtron SE, Dornkaulstr. 2, 52134 Herzogenrath, Germany

**Keywords:** Electronic devices, Electrical and electronic engineering

## Abstract

This work reports the epitaxial growth of 8.5 µm-thick GaN layers on 200 mm engineered substrates with a polycrystalline AlN core (QST by QROMIS) for CMOS compatible processing of vertical GaN power devices. The epitaxial stack contains a 5 $\upmu$m thick drift layers with a Si doping density of 2 × 10^16^ cm^−3^ and total threading dislocation density of 4 × 10^8^ cm^−2^. The thick drift layer requires fine-tuning of the epitaxial growth conditions to keep wafer bow under control and to avoid the formation of surface defects. Diode test structures processed with this epitaxial stack achieved hard breakdown voltages > 750 V, which is shown to be limited by impurity or metal diffusion from the contact metal stack into threading dislocations. Conductive Atomic Force Microscopy (cAFM) reveals some leakage contribution from mixed type dislocations, which have their core structure identified as the double 5/6 atom configuration by scanning transmission electron microscopy images. Modelling of the leakage conduction mechanism with one-dimensional hopping conduction shows good agreement with the experimental data, and the resulting fitting parameters are compared to similar findings on silicon substrates. The outcome of this work is important to understand the possibilities and limitations of vertical GaN devices fabricated on large diameter wafers.

## Introduction

As silicon devices reach their physical limits, wide bandgap materials, such as GaN and SiC, have been extensively studied and adopted by both academia and industry during the past decade. GaN power devices are today either the gold standard or, at least, a competitor that coexists with SiC technologies for certain applications up to 650 V^1,2^; thanks to an unprecedented capability of achieving low ON-state resistance (R_ON_) and high breakdown voltages (V_BD_). Therefore, a lot of research has been dedicated in the past few years to extend the range of applications for GaN devices to higher voltages. Vertical architectures are promising for reaching breakdown voltages of 1200 V and beyond, such as in the trench gate (semi) vertical MOSFET architecture^2,3^.

The first challenge in developing commercially viable technologies with GaN is the choice of substrate. While native GaN substrates offer the best material quality and thus better OFF-state characteristics, they can be prohibitively expensive, and they are only available on small wafer diameters up to 4 inches. Therefore, the fabrication of GaN devices on different types of substrates has been extensively explored^4^. In the present study, the fabrication of semi-vertical diodes with 5 µm thick drift layers on 200 mm wafers is reported for the first time. This was enabled by the growth of GaN epitaxial layers on QST substrates, which have a poly-AlN core that matches the coefficient of thermal expansion (CTE) of GaN^5^. This type of substrate offers the possibility of growing thicker drift layers due to excellent mechanical strength compared to Si substrates of the same size.

A contact metal stack previously developed for High Electron Mobility Transistors (HEMT) by imec has been shown to effectively reduce Rc by including and interfacial layer of amorphous Silicon (a-Si)^6^. However, it was not clear if this stack would be suitable for the vertical device architecture. This is because, as reported by some studies, metal diffusion from the contact stack, possibly by means of threading dislocation (TD) decoration^7,8^, is a potential limitation for using certain materials to achieve low contact resistances (Rc) in GaN. This is particularly critical vertical devices due to the presence of a blocking p/n^−^ junction below the contact. We address this issue by comparing two different ohmic contacts to n-GaN and the dependency of their performance on material quality and contact-to-junction distance.

Physical analysis found in literature has yielded conflicting results when it comes to identifying the role of each type of TD on the leakage current. While it is generally accepted today that screw type dislocations are mainly responsible for the leakage current^9–11^, cAFM has identified the mixed-type dislocations as possible contributor to the leakage current in some studies^12,13^. This type of dislocation has also been observed to result in nonradiative recombination centers^14^. Since this behavior is highly dependent on the core structure of the dislocations, and thus on the epitaxial growth conditions and the background doping, their characterization, especially when considering novel substrates, is of utmost importance. While several conduction mechanisms have been identified on GaN diodes, such as Trap-Assisted Tunneling, Space-Charge-Limited Conduction, Poole–Frenkel emission, lateral leakage ^15,16^ and hopping mechanisms (one or three dimensional)^17–19^, it remains unclear how they relate to the material properties. Hopping mechanisms, in particular, have been observed for different epitaxial growth techniques and substrates, but there’s a lack of systematic comparison between the extracted parameters and their effect on the leakage current.

This paper is organized as follows. “Introduction” describes the methods and tools used in this study, while “Methods” presents the overall characteristics of the epitaxial layers and the processing of the diodes used as test structures. The results are presented in “Results and discussion”, which is divided into three parts. “Growing thicker drift layers on 200 mm QST wafers” discusses the fine tuning of process conditions and the resulting material properties, “Junction leakage, breakdown and role of contact metal stack” shows the electrical results, namely, the leakage current and the breakdown characteristics of the diode test structures as well as their dependency on the contact metal stack. “Analysis of leakage conduction mechanism” presents a comprehensive analysis of the corresponding leakage mechanism from the diode test structures and compares it with similar findings from literature. The results and discussion are summarized in the conclusion section.

## Methods

The epitaxial layers were grown on 200 mm QST wafers in an AIXTRON G5+ C Planetary Reactor in 5 × 200 mm standard configuration, in which Ar was used in purge lines above the ceiling and below the base modules as an additional handle for reducing the wafer bow by minimizing the vertical thermal gradients. Measurements by means of cAFM were carried out on the Bruker ICON-PT tool in the Ar-filled glovebox and current maps were obtained with 1 pA sensitivity using in-house highly doped diamond tips. Here, voltage bias is applied to the sample and the current amplifier is connected to the probe. STEM imaging of the core structure of TDs were correlated to the cAFM measurements by imaging the same regions of the samples. Electrical measurements were performed with the Keysight B1505A Power Device Analyzer. Analytical modelling of data from electrical characterization of leakage currents was also performed.

### Epitaxial stack and processing

**Figure 1:**
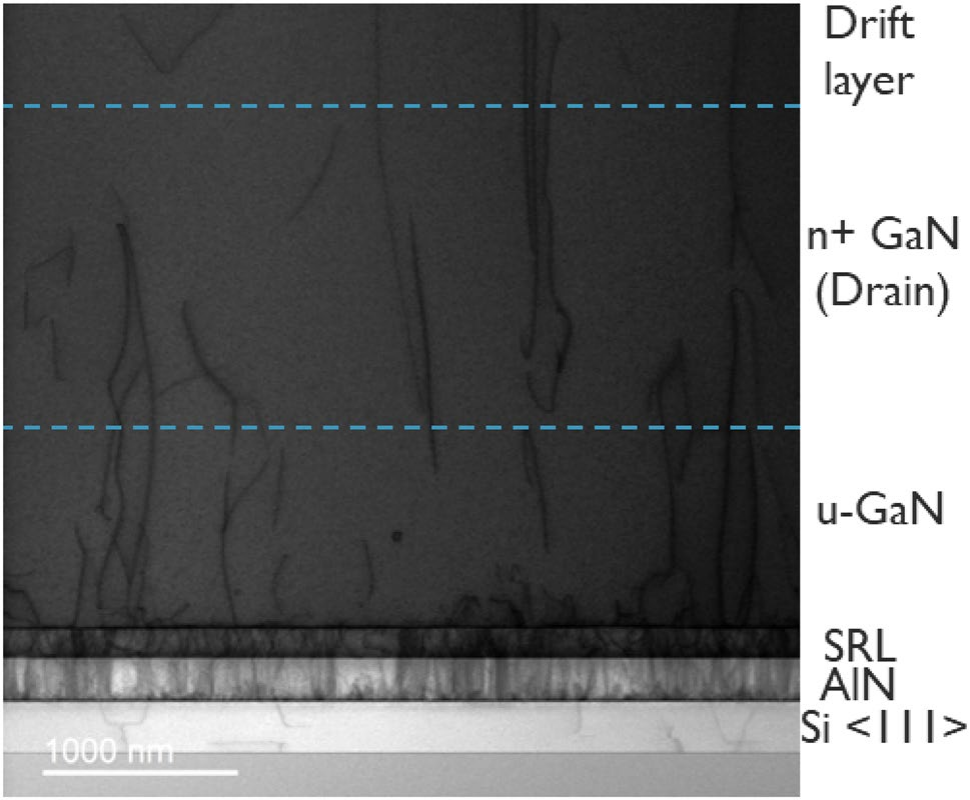
Cross-sectional SEM of Gen II epitaxial layers grown on a QST substrate.

Wafers of 200 mm diameter with poly-AlN core (QST) were chosen as the starting substrate since this material is CTE-matched to GaN, which are commercially available from QROMIS Inc. H_2_ ambient was used prior to the growth to clean and prepare the top Si <111> surface that is attached the poly-AlN core. The cleaning step was succeeded by a 200 nm AlN nucleation layer, followed by the growth of strain relief layers (SRLs). The SRLs of Gen I epitaxy is based on superlattice layers. The SRLs of Gen II epitaxy are typical Al-containing layers to transition from AlN to GaN. The GaN epitaxy is performed on top of the SRLs, starting with an unintentionally doped-GaN layer of 1 µm, used to tune wafer bow and warp while keeping the number of bevel particles under control. This layer also helps in terminating some of the dislocation lines before they reach the electrically active part of the device. The electrically active GaN layers are subsequently grown and have the following properties (from bottom to top): n^+^ GaN ([Si] 3 × 10^18^ cm^−3^) layer of 1.5 µm; 3 µm or 5 µm n^−^ GaN drift layer; 800 nm thick p-GaN ([Mg] from 2.5 × 10^18^ cm^−3^ to 1 × 10^19^ cm^−3^) and n^+^ GaN source layer ([Si] 5 × 10^18^ cm^−3^) of 200 nm. The total GaN thickness is 6.5 µm and 8.5 µm for stacks with 3 µm and 5 µm drift layers, respectively. The development of thicker and high quality epitaxial GaN layers can be divided into two distinct phases, referred to as Gen I and Gen II. These two epitaxial stacks differ in terms of stress compensation layer design and Si doping in the drift layer ([Si] 4 × 10^16^ cm^−3^ on Gen I and 2 × 10^16^ cm^−3^ on Gen II). The tuning of the SRLs on Gen II allowed the growth of 5 µm drift layers, which was not possible with the previous generation (discussed in “Growing thicker drift layers on 200 mm QST wafers”). Table 1 summarizes the thickness and doping of each GaN layer and a cross-sectional transmission electron microscopy (TEM) image is presented in Fig. 1. It is observed on Fig. 1 that most dislocation lines are terminated at the SRL/u-GaN interface and that a fewer number of them reach the n^+^ GaN region.

**Table 1:** GaN epitaxial layers description.

Region	Doping (cm^−3^)		Thickness (µm)	
	Gen I	Gen II	Gen I	Gen II
Top n^+^ GaN	5 × 10^18^	5 × 10^18^	0.2	0.2
p-GaN	1 × 10^19^	1 × 10^19^	0.8	0.8
n^−^ GaN (drift)	4 × 10^16^	2 × 10^16^	3	3 or 5
Bottom n^+^ GaN	3 × 10^18^	3 × 10^18^	1.5	1.5
u-GaN	-	-	1	1
Total			6.5	6.5 or 8.5

**Figure 2:**
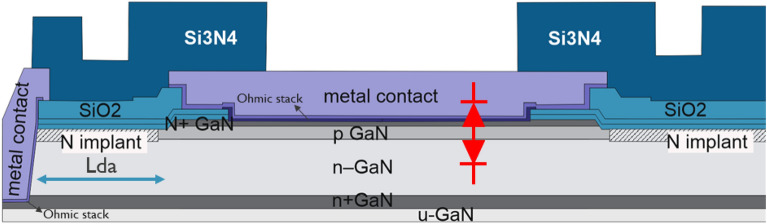
Schematic representation n^+^/p/n^−^ back-to-back diode structure.

The drift layer, which needs to accommodate the targeted breakdown voltage, forms a p/n^−^ junction with the p-body layer in this architecture. This junction should ultimately define the voltage blocking capability of devices processed with this stack. Additionally, the top n^+^ layer forms with the p-body layer a second, n^+^/p junction. As a result, a back-to-back diode structure can be identified between the top n^+^ layer and the buried n^+^ layer, as depicted in Fig. 2. A shallow mesa etch and nitrogen ion implantation are performed to ensure proper isolation of the test structures as well as a termination for the p/n^−^ junction.

The tested structures are two-terminal devices with a top and a bottom contact. During experiments, positive bias is applied to the bottom contact, which places the p/n^−^ junction in reverse bias and the top n^+^/p junction in forward bias. Besides the top contact to the n^+^ layer, a variation in which the top contact reaches the p-body layer is also processed by etching the top n^+^ GaN in the contact region. In the semi-vertical architecture, the buried n^+^ layer is contacted by means of a deep-via etch from the top wafer-surface. A Ti/Al/TiN based metal stack is used to form ohmic contacts to the n^+^ GaN layers and a low-temperature ohmic contact anneal between 500 and 600 °C is applied. The same process was used to form the top contact to the p-body layer, although it does not result in an ideal low-resistive ohmic contact. An optimized ohmic contact metal stack for reducing Rc that includes an interfacial thin layer of amorphous silicon (a-Si, deposited on both the buried n^+^ GaN and the top n^+^ GaN contacts when present) has been developed in previous studies on lateral GaN HEMTs^6^, which is considered here as a process variation. In the semi-vertical architecture presented here, the a-Si layer resulted in a reduction of 40% of Rc, but as further elaborated in the next sections, it was found to be detrimental for the off-state performance. SiO_2_ was deposited by plasma-enhanced chemical vapor deposition (PECVD) at different stages during the device processing as inter-layer dielectric (ILD). Lastly, as a final dielectric layer, 2 µm-thick Si_3_N_4_ is deposited which is opened at the end of the process flow in the bond pad areas of the diode structure.

## Results and discussion

### Growing thicker drift layers on 200 mm QST wafers

**Figure 3:**
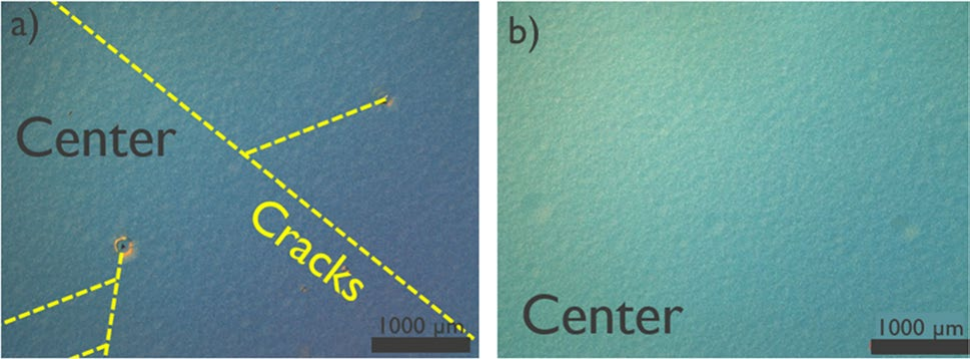
OM inspections for vertical GaN stacks grown on (**a**) generation I and (**b**) generation II QST substrates.

**Figure 4:**
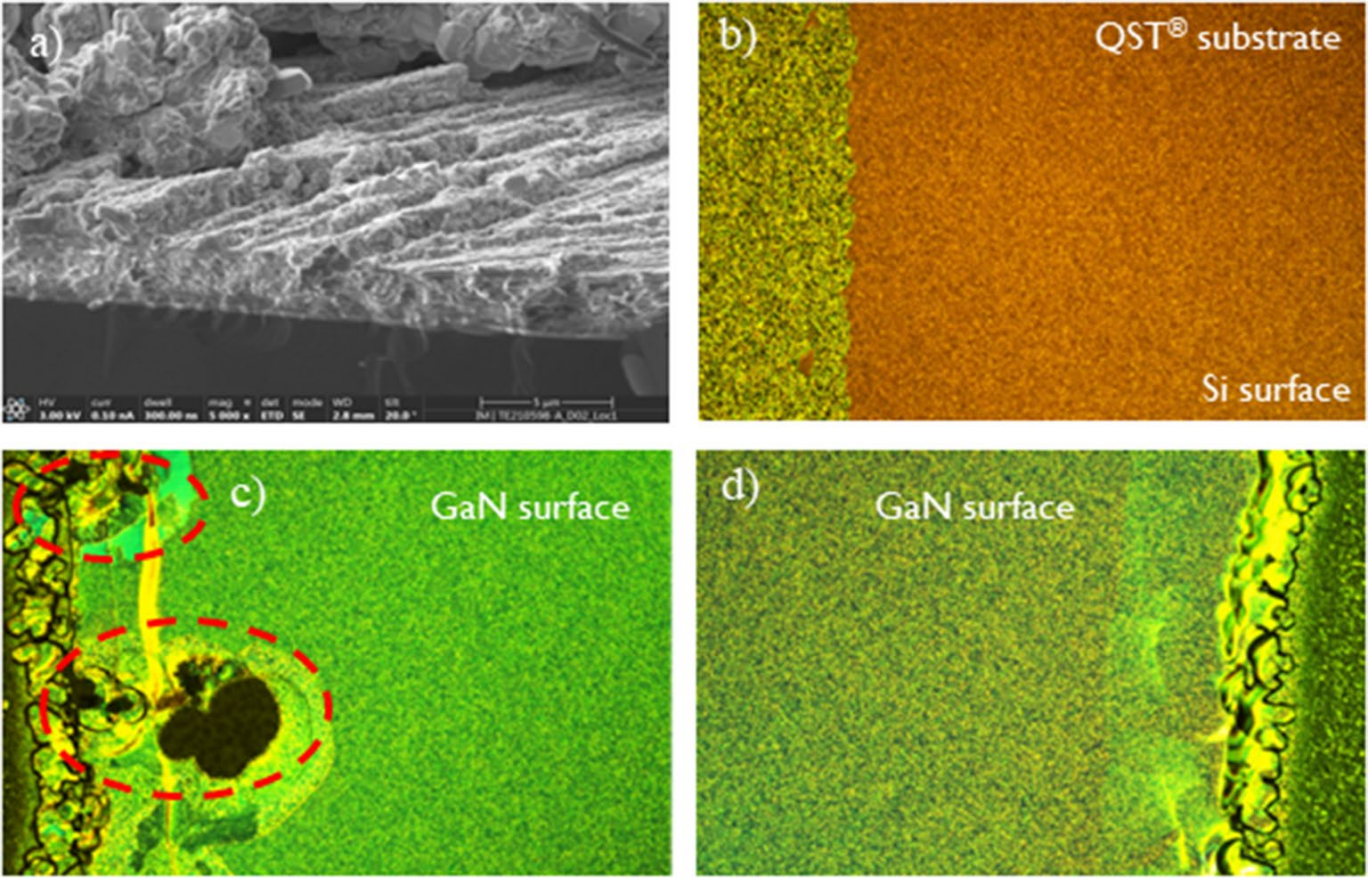
Identification and suppression of bevel particles on GaN surface. (**a**) TEM image identifying the bevel particles as amorphous or polycrystalline GaN; (**b**) initial substrate surface (Si <111> on top); (**c**) GaN surface with bevel particles after epitaxy using higher growth temperature for the buried n^+^ GaN layer and (**d**) Particle free GaN surface after epitaxy using lower growth temperature for the buried n^+^ GaN layer.

The SRL implementation from Gen I substrates was based on a complex superlattice scheme primarily to compensate for high convex bow of the incoming substrates. However, incoming wafers from Gen II exhibited flatter bow and thus were more suitable for the simpler AlGaN interlayer implementation. Figure 3.a and b show optical microscope (OM) images of the GaN surface at the center of wafers from Gen I and II, respectively after epitaxial growth with 3 µm drift layers. GaN stacks from Gen I present cracks and defects whereas nothing as such was observed for GaN stacks grown on Gen II epitaxy. Despite the better mechanical robustness of Gen II, further tuning is necessary to consistently reach final wafer bow with 5 µm drift layers within specification for device processing. We consider that the bow is within specification for processing in the 200 mm production line if its magnitude is less than 50 µm. Wafer bow could be further controlled by changing the Al percentage in the SRLs and by tuning the pressure during the growth of the u-GaN layer. Moreover, poly and amorphous GaN bevel particles were initially observed on Gen II wafers with 5 µm drift layers, identified as such by means of the TEM image depicted in Fig. 4a. Figure 4b shows one edge of the wafer surface prior to the epitaxial growth, in which the interface between the SiO_2_ and the top Si layer of the engineered substrate can be seen. Figure 4c, d show the wafer surface at its edge after epitaxy, before and after reducing the growth temperature of the botom n^+^ GaN, respectively. It can be seen that this modification was effective in suppressing the bevel particles at the edges of the wafer. However, understanding the root cause behind the origin of the bevel particles requires further investigation.

The improvement in crystal quality is demonstrated by the extracted Full Width at Half Maximum (FWHM) from the X-Ray Diffraction (XRD) peak shown in Table 2, and by the estimated concentration of screw and edge type dislocations shown in Table 3 obtained by a licensed algorithm that estimates the TD density from the XRD data. Both tables demonstrate the better material quality and uniformity of TD distribution in Gen II epitaxy. A reduction of 18% and 35% on the XRD FWHM <102> peaks was observed at the center and edge of the wafer, respectively, and 53% and 33% lower estimated concentration of screw type TDs and total TD density, respectively at the center.

**Table 2:** GaN <102> and <002> XRD FWHM peaks.

Gen	<102> (arcsec)			<002> (arcsec)		
	Center	Half	Edge	Center	Half	Edge
I	485	630	680	280	260	250
II	400	405	440	220	222	245

**Table 3:** Estimated concentration of edge and screw type threading dislocations from GaN stacks from Gen I and II QST substrates.

Gen	Edge type (× 10^8^cm^−2^)			Screw type (× 10^7^cm^−2^)		
	Center	Half	Edge	Center	Half	Edge
I	6	10	12	7.5	6.5	6
II	4	4.25	4.8	4.5	5	5.5

Hall measurements on test samples were performed to evaluate the net donor concentration and the electron mobility in the drift layers, with thickness of 3.75 µm and 4.5 µm for generations I and II, respectively, on substrates with GaN stacks grown without the top and bottom n^+^ GaN and p-GaN layers. As shown in Table 4, both have very similar net donor concentrations, even after the Si doping was reduced from 4 × 10^16^ cm^−3^ to 2 × 10^16^ cm^−3^ in Gen II. This was possible thanks to the greater flexibility for tuning growth parameters on Gen II, which allowed the reduction of the background carbon concentration to below 1 × 10^16^ cm^−3^. An increase in electron mobility of 36% and 185% was observed at the center and edges of the wafer, respectively, which further demonstrates the better material quality and uniformity of Gen II epitaxy following the trends seen in Tables 2 and 3.

**Table 4:** Net donor concentration and mobility within the drift layer for epitaxy generations I and II from Hall measurements.

Gen	Drift thickness (µm)	Net donor concentration (cm^−3^)	Mobility (cm^2^/V s)	
			Center	Edge
I	3.75	1.5 × 10^16^	471	227
II	4.5	1.6 × 10^16^	641	648

**Figure 5:**
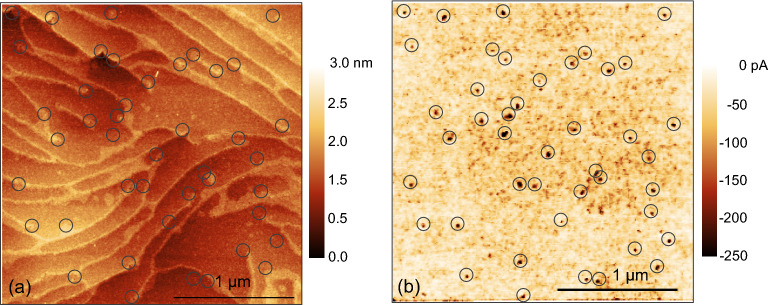
(**a**) Topography and (**b**) the corresponding current map obtained at a sample bias of −8 V of the p-GaN layer.

In Fig. 5.a, AFM topography on a test sample with exposed top p-GaN is shown; as the pGaN layer is sufficiently resistive, preferential conduction through dislocations can be seen in a cAFM current map (Fig. 5b). This sample exhibits a typical step-terrace morphology on which only screw and mixed-type threading dislocations are expected to interact and terminate the terraces^12,20^. These dislocations are encircled in black. The current map obtained at −8 V of sample bias in same area shows an enhanced local conductivity (dark dots) at the same dislocation locations as in Fig. 5a. Here, it is not possible to see pure-edge type dislocations in the topography, likely due to their small opening at the surface in comparison to mixed- and screw-type dislocations^13^.

**Figure 6:**
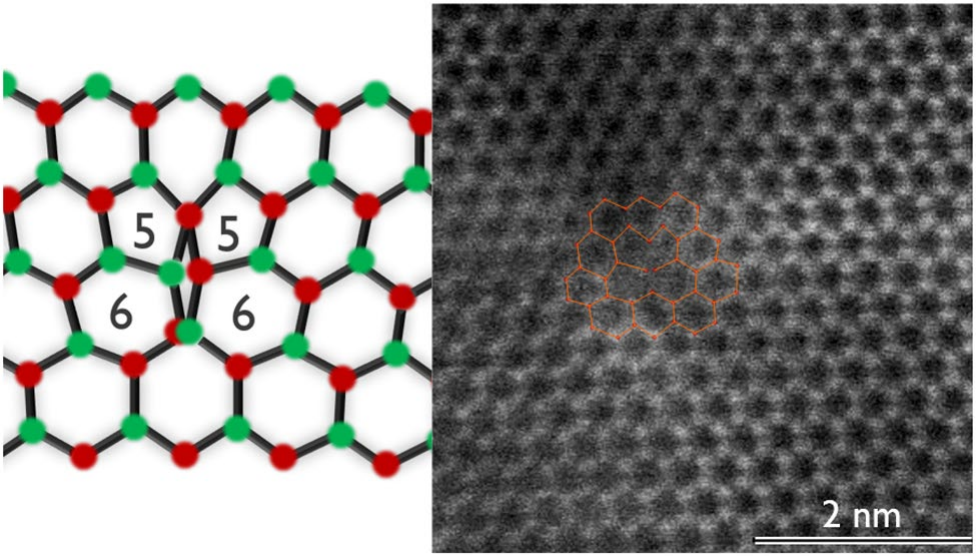
STEM image of undissociated mixed-type dislocation.

The most stable undissociated core configuration for the mixed type of dislocation is the double 5/6-atom ring core^21^, which introduces energy levels throughout the bandgap similar to pure screw type TDs. This means this core structure might be responsible for some leakage^12^ and non-radiative recombination^14^. Indeed, as verified by the STEM image shown in Fig. 6, the undissociated mixed-type dislocations have a double 5/6-atom ring core which correlates well with the results from cAFM imaging. Screw-type dislocations could not be found by STEM and correlated to the cAFM images due to their much lower concentration. However, as described in multiple reports ^9–11,20^ these dislocations should be the main cause for the leakage in GaN devices.

### Junction leakage, breakdown and role of contact metal stack

**Figure 7:**
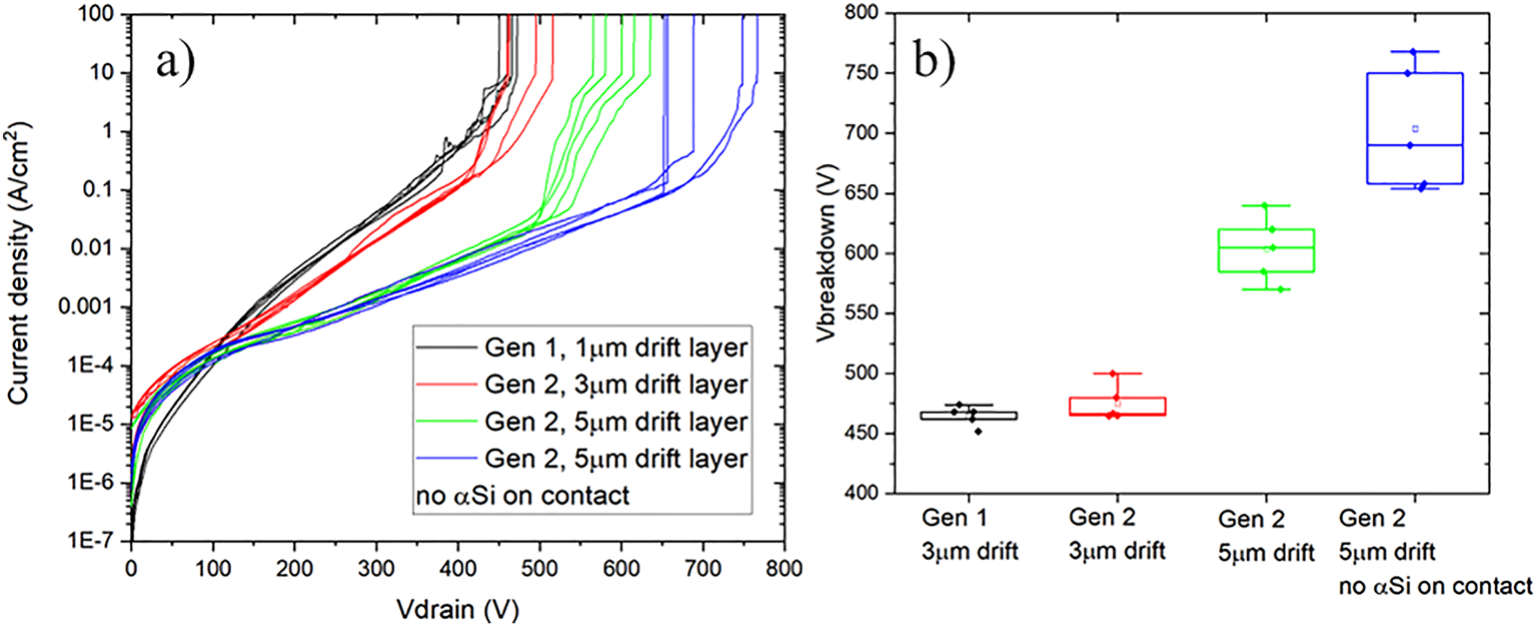
Back-to-back diodes reverse leakage and breakdown. (**a**) Current–voltage characteristics and (**b**) breakdown voltage for different samples.

Figure 7a depicts the current density as a function of the applied voltage of back-to-back diodes from Gen I and II, the latter with 3 µm or 5 µm drift layer and different metal stacks for the upper contact. Five nominally identical structures were measured on each wafer. Comparing samples with 3 µm drift layer from Gen I and Gen II, the latter results in slightly lower reverse leakage current and higher V_BD_ of 480 V, as shown in Fig. 7b. Thanks to the process optimization of Gen II, vertical GaN stacks with 5 µm drift layers became feasible, and resulted in lower leakage current and higher V_BD_ of ~ 605 V. All the diodes considered so far feature an interfacial layer of a-Si on the top contact metal stack. On the sample without the a-Si, V_BD_ reaches an average of 700 V, with some back-to-back diodes reaching ~ 800 V, which is about 80% of the theoretical breakdown voltage with this drift layer thickness and Si doping, considering an ideal structure and critical electric field of 2.5 MV/cm as the onset of impact ionization.

**Figure 8:**
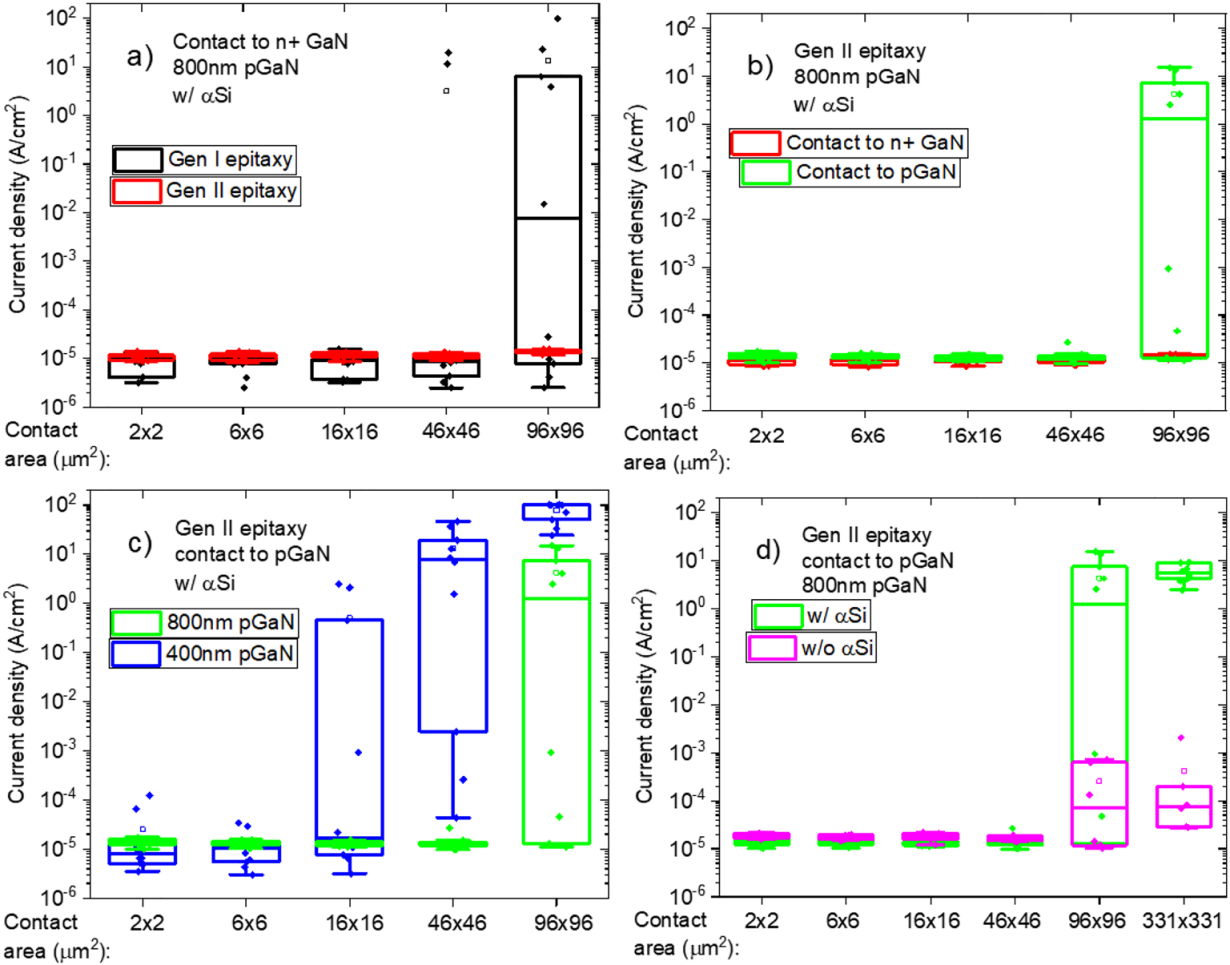
Current density at 20 V with scaling of contact area. (**a**) Generation I versus generation II epitaxy; (**b**) contact to n^+^ GaN versus contact to p-GaN; (**c**) 800 nm versus 400 nm p-GaN; (**d**) contact with αSi versus no αSi.

Besides an impact on the breakdown voltage, a reduction of yield on these test structures with the inclusion of a-Si on the contact stack has been observed. Figure 8 depicts the current density at an applied voltage of 20 V for diodes with different top contact areas, in which the data from 10 identical structures is shown for each case. In Fig. 8, the contact area is scaled, while the device dimensions are kept constant (the active area is 100 × 100 µm^2^), except for the device with the largest contact in Fig. 8d (the active area is 335 × 335 µm^2^). Figure 8a compares the current density of back-to-back diodes fabricated on Gen I and Gen II substrates. Some loss of yield (that is, very high current densities at low voltages) can be seen on diodes from Gen I with 46 × 46 µm^2^ contact area, which becomes even more critical with bigger contacts. However, diodes on Gen II substrates, which have better crystal quality, presented no such issue with contacts of dimensions up to 96 × 96 µm^2^. This relation between yield, contact area and epitaxy implies that decoration of TDs with metal or impurities from the contact stack is possible. To test this hypothesis, vertical diodes with contact to p-GaN were also measured with varying contact area. Figure 8b shows the comparison between diode structures from Gen II with contacts to the n^+^ GaN and p-GaN layers, in which the yield issue is seen again on diodes with 96 × 96 µm^2^ area with contact to p-GaN. This is possibly because with the deeper contact to p-GaN, the contact stack is closer to the p/n^−^ junction, thus, the metal needs to diffuse through a shorter distance to short this junction. This can also be seen on Fig. 8c, which compares vertical diodes with contact to p-GaN layers with thicknesses of 800 nm and 400 nm. Loss of yield is already seen on 16 × 16 µm^2^ contacts on the wafer with 400 nm thick p-GaN, which once again fits within the hypothesis of metal decoration of TDs since in this case the metal stack is again closer to the p/n^−^ junction. Figure 8d compares vertical diodes with and without a-Si as an interfacial layer in contacts with areas up to 331 × 331 µm^2^. The yield issue is less critical, if not vanishing, without a-Si, even though some increase in leakage can still be seen from contact areas of 96 × 96 µm^2^.

The following mechanism can be proposed to explain these observations regarding breakdown voltage and yield. During the ohmic annealing step, some metal or silicon from the contact stack diffuses into TDs. In some of them, the impurity diffusion reaches depths greater than 1 µm^8^, which would then short the p/n^−^ junction rendering it ineffective. The bigger the contact area, the greater the chance is of decorating one or more TDs with metal or silicon up to this depth. Similarly, with higher TD density the junction is more prone to being shorted for a given contact area (Fig. 8a). This is also true if the contact is placed deeper and closer to the p/n^−^ junction as in Fig. 8b, c. As described by Brice De Jaeger et al*.*^6^, the a-Si reduces the melting point of Al and enhances its diffusion. If diffusion into TDs is also enhanced, this may account for the trend seen in Fig. 8d. It is possible that some metal diffusion happens even without a-Si since somewhat higher leakage can still be seen with bigger contacts. Moreover, even if the metal diffusion does not go deep enough or if it does not completely short the p/n^−^ junction, it might weaken its blocking capability resulting in lower breakdown voltages when employing a-Si, as observed in Fig. 7. Even though the a-Si layer in the ohmic contact stack is effective in reducing the contact resistance, it has been demonstrated that it could diffuse, or enhance diffusion of other species, along threading dislocations down to the main p/n^−^ junction, limiting the blocking capabilities of vertical p/n^−^ junctions and in some cases causing severe yield losses.

### Analysis of leakage conduction mechanism

**Figure 9:**
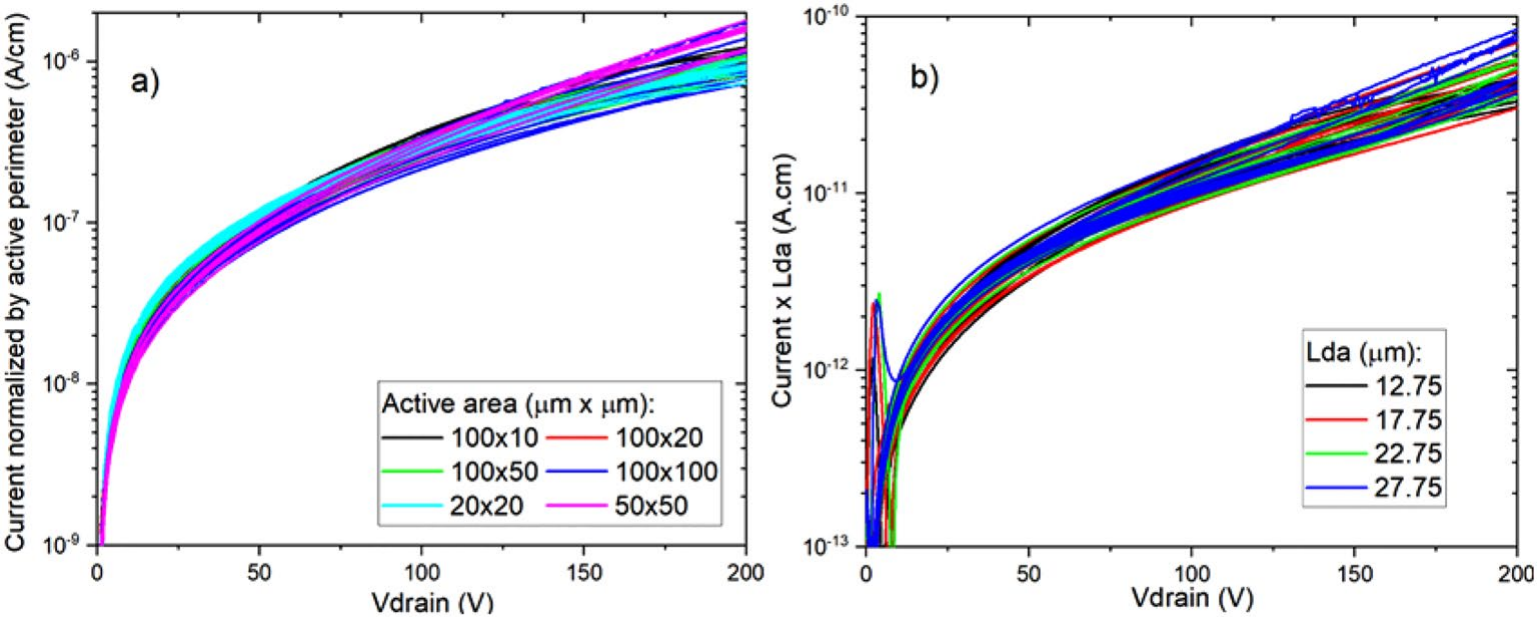
Current scaling on back-to-back diodes up to 200 V of reverse bias. (**a**) Current normalized by active perimeter and (**b**) current scaled by deep via to active distance (Lda).

Figure 9a shows the current density normalized by the active device perimeter as a function of the voltage of back-to-back diodes with varying active dimensions on Gen II substrates with 5 µm drift layer as measured on 10 dies. It can be seen that for voltages up to 200 V, the current is proportional to the perimeter of the diode structures, since the curves overlap after normalizing the current by the active perimeter. This indicates that the leakage current in this bias range is generated either on the sidewalls of the N-implanted isolation region surrounding the active area or on the etched mesa possibly due to etch damage. In Fig. 9b, the current has been scaled by deep via to active distance (Lda, see Fig. 2) as Current x Lda, showing that the current also scales with this parameter and thus flows on the horizontal direction.

From voltages above 300 V and up to breakdown, the current of the back-to-back diodes scales with the active area, which indicates vertical current flow through the p/n^−^ junction. The voltage range between 200 and 300 V is a transition from lateral to vertical dominant leakage. To gain more insight on how the material properties affect the leakage current, different conduction mechanisms were considered aiming to fit the I–V characteristics. Very good agreement between analytical calculations and experimental data was achieved with the 1D-hopping (1DH) conduction mechanism^17–19^, whereas fitting with other conduction mechanisms was attempted, but did not yield good agreement with experiments. The identification of 1DH rather than variable range hopping (VRH) as the main leakage conduction mechanism indicates conduction through TDs lines rather than bulk defectivity hopping^19^.

Equation (1) is the 1DH analytical expression for the current density^17^,

1 $$J=\frac{{N}_{td}q{v}_{0}}{1+\frac{2\mathrm{exp}\left(\frac{q{E}_{\sigma }}{kT}\right)}{\mathrm{exp}\left(\frac{qb{E}_{av}}{kT}\right)-1}}$$

in which kT/q is the thermal voltage. The electric field E_av_ was computed as the average electric field on the drift layer after full depletion, a reasonable assumption for voltages above 200 V, using the formula ${E}_{av}=(V+{\varphi }_{bi})/{t}_{drift}$. In this formula, ${\varphi }_{bi}$ is the built-in potential of the p/n^−^ junction (~ 3.3 V) and ${t}_{drift}$ is the drift layer thickness.

Equation (2) can be used to calculate the average trap energy of electrically active defects created by TDs^17^ if one assumes that hopping through bulk defects is negligible in comparison to 1DH.

2 $${v}_{0}={\Gamma }_{1}{e}^{\frac{-2b}{\boldsymbol{\hbar }}\sqrt{2{m}^{*}Et}}$$

${\Gamma }_{1}$ in Eq. (2) is the attempt-to-hop frequency (we use 10^13^ Hz as in other studies^17,22^), ${m}^{*}$ is the electron effective mass in GaN (0.2 ${m}_{0}$) and $\boldsymbol{\hbar }$ is the normalized Planck constant. The definitions of each parameter are summarized in Table 5, along with corresponding units.

**Table 5:** Definition and units of one-dimensional hopping fitting parameters.

Parameter	Definition	Fitted value		Unit
		@25 °C	@150 °C	
${N}_{td}$	Threading dislocation density (pure screw type)	4.5 × 10^7^		cm^−2^
$b$	Hopping distance	2	2.7	nm
Et	Trap energy level from Ec associated to dislocations	0.24	0.21	eV
${v}_{0}$	Carriers hopping frequency	10^11^	3 × 10^10^	Hz
${E}_{\sigma }$	Width of defect subband	0.265		eV

As demonstrated by the cAFM analysis depicted in Fig. 5, both mixed and pure screw TDs result in leakage paths through the buffer. These two kinds of TDs have been linked to leakage currents on sapphire substrates also through cAFM^12,13^ and to the formation of strong non-radiative centers in free-standing GaN^14^. However, pure screw type TDs have been identified as the main contributors to the leakage current on p/n and Schottky GaN diodes^23,24^ (especially the ones with full core structure, as recently demonstrated in references ^12,20^). Thus, we use the pure screw type TD density estimated from XRD for the fitting of 1DH conduction mechanism (${N}_{td}$ = 4.5 × 10^7^ cm^−2^). It is important to note that it is possible to fit the experimental data considering conduction through mixed type dislocations as well, which requires a certain dislocation density ${\mathrm{N}}_{\rm{td}}$ between around 35% and 90% of the total TD density^14,25,26^ and minor changes to b and ${E}_{\sigma }$. Thus, one cannot unambiguously determine if 1DH conduction is mainly through mixed or pure screw dislocations just by means of fitting Eq. (1).

**Figure 10:**
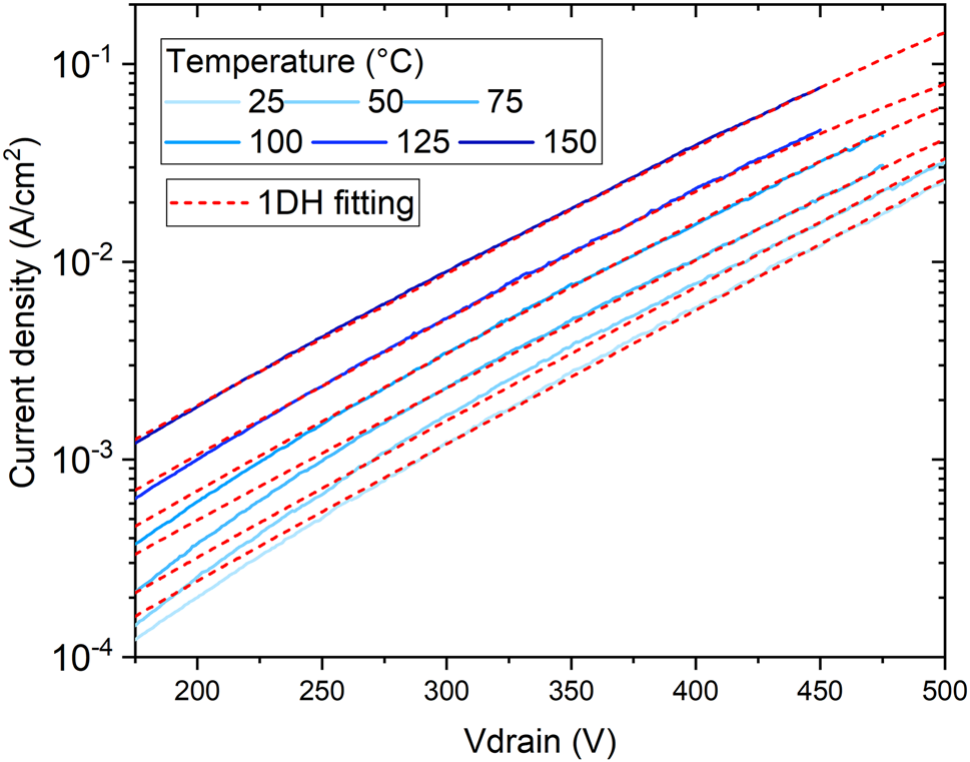
Current density as a function of the applied voltage from 25 to 150 °C of back-to-back diodes and one-dimensional hopping conduction mechanism fittings.

From the slope of Eq. (1), *b* can be determined. Increase in slope with increasing temperature was observed on the experimental data, which can be seen on Fig. 10 along with the corresponding fitting lines for different temperatures. The extracted values of b ranged from 2 to 2.7 nm (at T = 25 °C and 125 °C, respectively), possibly due to the increase on the lateral current with temperature at a different rate and the reduction of the onset voltage of the 1DH mechanism. At room temperature, ${v}_{0}={10}^{11}$ Hz is used as in previous reports^17,18^, from which Et = 0.24 eV was obtained. At higher temperatures, ${v}_{0}$ is calculated from Eq. (2), initially considering the value for Et extracted at room temperature. From the obtained values for b, ${E}_{\sigma }$ can be obtained by fitting Eq. (1) to the experimental IV characteristics, which results in ${E}_{\sigma }$ = 0.265 eV. If one considers that the average trap energy level Et can slightly shift to shallower levels with increasing temperature, this calculation can be reiterated to fine tune ${v}_{0}$. This procedure yields values for ${v}_{0}$ from 10^11^ Hz to 3 × 10^10^ Hz and for Et from 0.24 to 0.21 eV when the temperature is increased from 25 to 125 °C.

**Figure 11:**
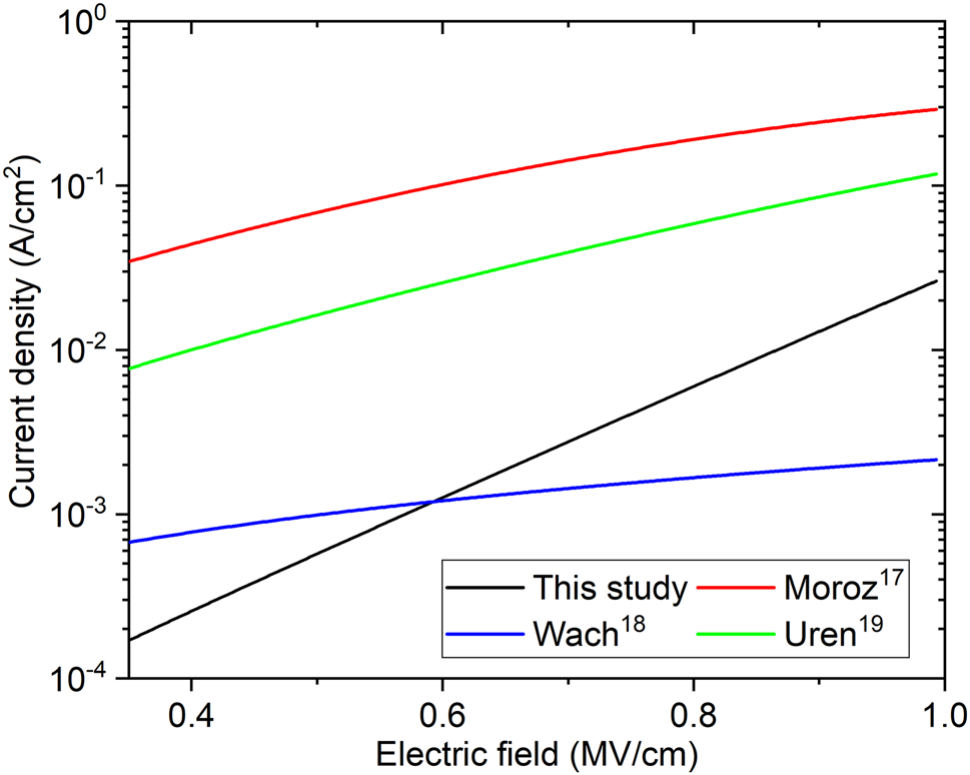
One dimensional hopping analytical calculation and comparison to other studies.

Table 6 shows a summary of the fitting parameters obtained in this study as well as the ones from previous studies on Si substrates at room temperature. Figure 11 compares the analytical models using these parameters and Eq. (1) as a function of the electric field from 0.35 MV/cm (which corresponds to 180 V reverse bias in our stack). The pure screw dislocation densities extracted in other reports^17,19^ were similar to the ones in the present study. As discussed in Uren et al*.*^18^, the actual ${N}_{td}$ in their epitaxy should also be close to 10^9^ cm^−2^, but since conduction through a defect band in the carbon-doped layer of their device was not included, ${N}_{td}$ = 10^5^ cm^−2^ was sufficient for the modelling considered there. Thus, for comparison purposes, we use ${N}_{td}$ matched to Moroz et al.^17^ in the calculation of Fig. 11. The narrower defect sub-band (${E}_{\sigma }$) in these studies results in current densities that are one or two orders of magnitude higher (except for the ones found in Wach et al*.*^19^ due to the lower ${v}_{0}$ used there). On the other hand, the higher b found here results in a steeper slope, so that the current density approaches the one found in Uren et al*.* and Moroz et al*.*^17,18^ at higher electric fields. While the same ${v}_{0}$ used in these two studies worked well to fit our experimental data, the higher hopping distance found here resulted in a shallower trap energy level (Et). Even though it is argued in Moroz et al*.*^17^ that Et = 0.85 eV matches the one linked to screw type TDs found in early studies^27^, it is important to realize that TDs result in multiple energy levels distributed all over the bandgap ^12,14,20,20,21,23,24^. The energy trap level Et, thus, represents a rough estimate of the average of these trap energy levels plus other possible trap states not associated to dislocations. The values for ${v}_{0}$ and b found in Wach et al*.*^19^ do not result in a reasonable value for Et, however, the modelling there yields current densities closer to the ones presented here, despite the lower slope due to the small b of 0.1 nm.

**Table 6:** One-dimensional hopping fitting parameters and comparison to other studies.

Parameter	This study		Moroz^17^	Uren^18^	Wach^19^
Substrate	Poly-AlN		Si		
Temperature	25 °C	150 °C	25 °C		
${N}_{td}$	4.5 × 10^7^		3 × 10^7^	10^5a^	6 × 10^8^
$b$	2	2.7	1.1	1.1	0.1
Et	0.24	0.21	0.85	0.85	–
${v}_{0}$	10^11^	3 × 10^10^	10^11^	10^11^	10^9^
${E}_{\sigma }$	0.265		0.08	0.12	0.06

^a^Not used in the calculation of Figure. 3 × 10^7^ cm^−2^ was used instead.

The proportion of each type of TD, as well as the configuration of their cores, is highly dependent on the epitaxial growth conditions^9^. Moreover, it has been reported that different types of TDs can capture and diffuse impurities. TDs have been linked to vacancy clustering^28^ and Mg diffusion has been experimentally observed in both edge type^29^ and mixed type^30^ TDs. Therefore, it is expected that the background doping of the location of the dislocation lines impacts the electrical properties of the resulting trap states. These observations might then explain the shallower trap energy level, as well as the higher hopping distance and width of the defect sub-band found here. The studies conducted by Uren et al*.* and Wach et al*.*^18,19^ concerned AlGaN/u-GaN/Carbon doped GaN and, like in Moroz et al*.*^17^, the epitaxial growth was performed on Si substrates. In this paper, the first analysis of this kind on 200 mm poly-AlN wafers is presented, which required particular growth conditions that may result in substantially different electrical characteristics of TDs.

## Conclusion

This work presents the development of thick GaN epitaxial layers on 200 mm wafers with polycrystalline-AlN CTE-matched core to GaN (QST) and the relationship between material properties, leakage, and breakdown of diode structures on this type of substrates. GaN epitaxial growth containing 5 µm thick drift layers with 2 × 10^16^ cm^−2^ Si and total TD density of 4.5 × 10^8^ cm^−2^ was achieved. Optimized growth conditions result in lower TD density with improved uniformity across the wafer. Total TD density and screw type TDs were reduced by 33% and 53%, respectively, while electron mobility in the drift layer was increased from 471 cm^2^/(V∙s) to 641 cm^2^/(V∙s). Conductive-AFM shows the presence of leakage spots on mixed and screw type TDs, while the core of mixed type TDs could be identified as the double 5/6 configuration by TEM imaging. Moreover, by comparing stacks with different material quality, diode structures with geometrical variations and different ohmic metal contact schemes, a detrimental effect due to species diffusion through TDs reaching the p/n^-^ junction was discussed, highlighting the importance of the metal stack choice for vertical GaN devices. With the optimal epitaxy and process described in this work, diodes reaching 750 V of average breakdown voltage were demonstrated. Lastly, the fitting of one-dimensional hopping conduction model reveals the nature of the current transport, from which relevant parameters could be extracted and compared to previous findings in the literature for Si substrates and different growth conditions. These results are important to pave the way towards cost-effective manufacturing on large substrates of vertical GaN power devices with high breakdown voltages, and sheds light on the intricate relation between material properties and the leakage through the vertical stack.

## Data Availability

All data related to this study are available from the corresponding author upon reasonable request.
